# Gating with Charge Inversion to Control Ionic Transport in Nanopores

**DOI:** 10.1021/acsanm.2c03573

**Published:** 2022-12-01

**Authors:** Wilfred S. Russell, Chih-Yuan Lin, Zuzanna S. Siwy

**Affiliations:** † Department of Chemistry, University of California, Irvine, California 92697, United States; ‡ Department of Physics and Astronomy, University of California, Irvine, California 92697, United States; § Biomedical Engineering, University of California, Irvine, California 92697, United States

**Keywords:** nanopore, charge inversion, ion correlations, fluctuations, rectification

## Abstract

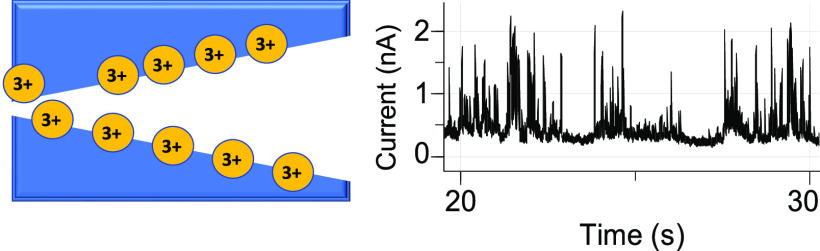

Multivalent ions modify the properties of the solid/liquid interfaces, and in some cases, they can even invert the polarity of surface charge, having large consequences for separation processes based on charge. The so-called charge inversion is observed as a switch from negative surface charge in monovalent salts, e.g., KCl, to effective positive surface charge in multivalent salts that is possible through a strong accumulation and correlation of the multivalent ions at the surface. It is not known yet, however, whether the density of the positive charge induced by charge inversion depends on the pore opening diameter, especially in extreme nanoconfinement. Here, we probe how the effective surface charge induced by charge inversion is influenced by the pore opening diameter using a series of nanopores with an opening between 4 and 25 nm placed in contact with trivalent chromium ions in tris(ethylenediamine)chromium(III) sulfate at different concentrations. Our results suggest that the effective positive charge density can indeed be modified by nanoconfinement to the extent that is dependent on the pore diameter, salt concentration, and applied voltage. In addition, the correlated ions can increase the transmembrane current in nanopores with an opening diameter down to 10 nm and cause a significant blockage of the current for narrower pores. The results provide guidelines to control ionic transport at the nanoscale with multivalent ions and demonstrate that in the same experimental conditions, differently sized pores in the same porous material can feature different surface charge density and possibly ion selectivity.

## 1 Introduction

Charge inversion is ubiquitous in nature. It is important in processes such as DNA condensation, viral packing, and colloidal coagulation.^1−8^ Charge inversion occurs when a charged surface comes in contact with a critical concentration of multivalent counterions that over-screen and invert the native charge. As an example, a surface with carboxyl or silanol groups becomes effectively positively charged when in contact with divalent calcium or trivalent cobalt ions.^7,9^ In nanopores, charge inversion induces a switch from cation selectivity in negatively charged nanopores with monovalent salts such as NaCl or KCl to anion selectivity using multivalent cations.^10^ Consequently, a cation exchange membrane when exposed to a solution containing multivalent cations can drastically change its function by becoming an anion exchange membrane.

The accumulation of counterions on the surface is possible through the spatial correlation of counterions that lowers the system’s potential energy and overcomes electrostatic repulsion.^1^ The adsorbed counterions have been postulated to form a strongly correlated liquid (SCL), like the structure of a Wigner crystal,^1,11^ as recently revealed under electron microscopy.^12^ Thus far, the experimental efforts have been focused primarily on using nanopores and nanochannels as a model system to identify conditions where overcharging occurs. Measurements of electrokinetic phenomena such as streaming current,^7^ open circuit potential,^10^ and current–voltage (*i*–*V*) curves^6,9,13−15^ have indeed identified the critical concentration needed to switch the sign of the effective surface charge. However, very little is known about how the onset of charge inversion influences ionic transport, especially in nanopores whose opening diameter is comparable to the size of the multivalent ions.

In this manuscript, we use conically shaped nanopores in polymer films^16−19^ as a model system to understand the role of charge inversion in tuning ionic transport under nanoconfinement. We used nanopores with opening diameters between 25 and 4 nm and tris(ethylenediamine)chromium(III) sulfate as the multivalent electrolyte that is expected to induce charge inversion. The trivalent tris(ethylenediamine)chromium(III) cation is bulky with a diameter of ∼0.9 nm, similar to the previously reported trivalent cobalt ion cobalt(III) sepulchrate (CoSep).^7,9,20^ Conically shaped nanopores are especially suited to probe charge inversion. Their *i*–*V* characteristics are sensitive to the sign and magnitude of surface charges.^21−27^

The main questions we wanted to address with this work are: How does charge inversion influence the conductance of nanopores with different opening diameters? Is it possible that when the pore opening diameter is 10 nm or less, the accumulation of trivalent ions blocks the ionic transport due to steric hindrance? Does this steric hindrance lead to a geometry-induced blockage that results in ion current instabilities and in turn modifies charge inversion? And finally is the effective charge density induced by charge inversion pore diameter dependent?

To answer the questions, we have divided the manuscript in the following sections. Section 3.1 describes the model system of conically shaped nanopores and is followed by the fundamentals of charge inversion, as explained by the SCL model^1,3^ in Section 3.2. Section 3.3 provides an answer to the first question on pore-diameter-dependent effects of charge inversion on ionic transport. Subsection 3.3.2 specifically discusses conditions when charge inversion could lead to ion current enhancement as well as transient blockages observed as conductance instabilities, while subsection 3.3.3 gives an example where a nearly complete shutoff of the current was obtained. Section 3.4 provides two modeling approaches that predict the dependence of the effective charge density induced by multivalent ions on pore diameter.

## 2 Materials and Methods

### 2.1 Chemicals

Tris(ethylenediamine)chromium(III) sulfate, Trizma base, minimum 99.9% titration, and poly(allylamine hydrochloride) (average MW ≈ 17,500) (PAH) were purchased from Sigma-Aldrich (St. Louis, MO, USA). Potassium chloride (KCl), formic acid, and sodium hydroxide (NaOH) were purchased from Fisher Scientific (Hampton, NH, USA). Double deionized (Milli-Q) water used throughout experimentation was purified to a resistivity of 18.2 MΩ using a Milli-Q IQ 7000 water purification system (MilliporeSigma, Burlington, MA, USA). All chemicals were used as received and not purified any further.

### 2.2 Conical Pore Fabrication and Characterization

Polyethylene terephthalate (PET) membranes with single conical nanopores were fabricated using the track-etch method described previously. Briefly, 3 cm in diameter, 12 μm thick PET, polymer foils (Hostaphan RH12 Hoechst) were first irradiated using a single, energetic (11.4 MeV/u) Au ion at the UNILAC (Universal Linear Accelerator) (GSI Helmholtz Centre for Heavy Ion Research, Darmstadt, Germany).^28^ In the next step, the films were UV irradiated for 1 h on each side (UVGL-25 Compact UV Lamp from UVP, LLC, Upland, CA, USA) and then subjected to chemical etching.^17^ To obtain conically shaped nanopores, the etching was performed from one side only whereby one side of the pore was in contact with 9 M NaOH, and the other side, with a 1 M formic acid and 1 M KCl (STOP).^17^ 1 V was applied during etching using a Keithley 6487 picoammeter/voltage source (Keithley Instruments, Solon, OH, USA). Measurable breakthrough of the transmembrane current indicated that the pore was etched through. Once etched, the pore was left overnight in STOP solution before being sized.

Prior to use, the diameter of each PET nanopore was electrochemically sized using a well-established, nondestructive characterization technique where the measured conductance in a solution of known conductivity was used to calculate the pore tip diameter.^17^ Electrochemical sizing measurements were performed with a Keithley 6487 picoammeter/voltage source (Keithley Instruments, Solon, OH, USA), and software written in-house on MATLAB (MathWorks, Natick, MA, USA). Using Ag/AgCl electrodes (in-house chlorinated Ag wire) as both the working and reference electrodes, the voltage was ramped from −0.1 to +0.1 V (10 mV steps) in unbuffered 1 M KCl. This potential window was chosen to ensure that a linear *i*–*V* curve was obtained for sizing. The measured pore conductance was used to calculate the pore tip diameter. The diameter of the base opening conversely was calculated based on the total etching time and the bulk etch rate: 2.13 nm/min.^17^ The method of measuring the tip of conical pores using *i*–*V* curves is well established in the literature. The tip diameter obtained from the electrochemical method was confirmed before with the resistive-pulse technique where individual molecules or particles pass through a pore and block the current to the extent that depends on both the pore and molecule/particle diameters.^28,29^ The 5 nm tip diameter of a conical nanopore was measured both electrochemically and with individual molecules of porphyrin.^30^ In another, independent approach, conductance of conical nanopores was probed in the presence of polyethylene glycol (PEG) molecules of different molecular weights and thus different diameters.^31^ The size of PEG molecules that led to a significant drop of the current served as an estimate of the pore opening that was in agreement with the diameter measured using the electrochemical method described above.^32^

Pores used for experimentation had opening tip diameters between 4 and 25 nm and the base opening diameters between 700 nm and 1 μm in size. The cone opening angle was less than 5°. Each conical nanopore was also characterized in 100 mM KCl, pH 8, as shown in Figure S1. These recordings were performed with a Keithley 6487 picoammeter/voltage source (Keithley Instruments, Solon, OH, USA). The voltage was ramped between −2 and +2 V with 200 mV steps, applying voltage for 2 s and recording the current value at the end of each step. The presented *i*–*V* curves are averages of three forward and reverse scans.

### 2.3 Modification of Pore Walls with PAH^33^

Once etched and sized, the PET nanopore was exposed to a 0.625 g solution of PAH dissolved in 5 mL, pH 6 Milli-Q water and left overnight to modify before being rinsed with Milli-Q water and used the next day for measurements. *i*–*V* curves as described previously^19,27^ were obtained to verify modification success.

### 2.4 Measurements of Ion Current in Time

Signals of ion current in time for each nanopore in contact with solutions of tris(ethylenediamine)chromium(III) sulfate were recorded with an Axon Instruments Axopatch 200B integrated patch clamp and 1322A Digidata acquisition system (Molecular Devices, LLC, San Jose, CA, USA). All ion current measurements were hardware low-pass Bessel filtered at 1 kHz (80 db/decade), sampled at a frequency of 20 kHz, and digitized at 10 kHz. pH 8 buffered solutions (low-conductivity Trizma base) containing 0.1, 1, and 10 mM tris(ethylenediamine)chromium(III) sulfate were used throughout experimentation. The pH of all the solutions was confirmed using a Fisherbrand accumet AB150 pH benchtop meter (Hampton, NH, USA). Each nanopore was subjected to all three concentrations of tris(ethylenediamine)chromium(III) sulfate in the order from the lowest, 0.1 mM, to the highest concentration, 10 mM, with thorough washing in deionized water when changing solutions. The electrolyte concentration was the same in both chambers of the conductivity cell. Ion current was recorded using episodic linear forward (−2 to 2 V; 200 mV steps) and reverse (2 to −2 V; 200 mV steps) potential sweeps, recording ion current time series at each voltage step for 50 s. The average and standard deviation of the time series in five forward and reverse sweeps were formed into *i*–*V* curves and used to probe the polarity of the surface potential of the PET nanopore. The entire apparatus was placed inside a Faraday cage (Warner Instruments, Hamden, CT, USA) on top of a vibration cancellation table (TMC, Peabody, MA, USA). The ion current in time series was analyzed using Clampfit 10.4 (Molecular Devices, LLC, San Jose, CA, USA).

## 3 Results and Discussion

### 3.1 Conically Shaped Nanopores as a Model System to Probe Diameter-Dependent Charge Inversion

**Figure 1:**
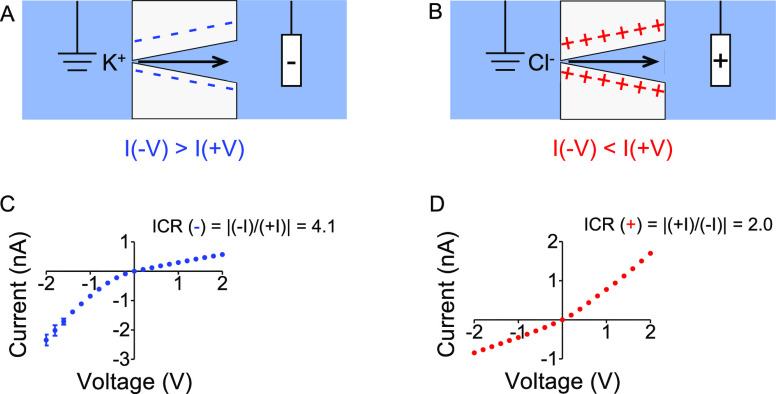
Probing surface charges in a conical nanopore by measuring *i*–*V* curves in KCl salt solutions. (A, B) Schemes of the conically shaped nanopore with negatively (A) and positively (B) charged pore walls. The arrows indicate the direction of cation (A) and anion (B) migration for the voltage polarity that produces higher currents. (C) Recordings for an as-prepared 4 nm small opening (tip) and a 720 nm wide (base) in diameter conical nanopore placed in 100 mM KCl. (D) Recordings in 100 mM KCl for the same nanopore as in (C) after it had been modified with PAH. Modification with PAH rendered the nanopore to be positively charged. All *i*–*V* curves are averages of three forward and reverse scans performed with a Keithley picoammeter/voltage source. The error bars were calculated by standard deviations of the three scans. Ion current rectification (ICR) was calculated based on currents at −2 and +2 V.

Single conically shaped nanopores in 12 μm thick PET films were prepared by the track-etching technique, which entails irradiation with single energetic Au ions (UNILAC, GSI Darmstadt, Germany), followed by asymmetric wet chemical etching in sodium hydroxide, as reported previously.^17,34^ This fabrication process leads to the formation of a highly dense carboxyl group network on the pore walls and membrane surface.^35^ As-prepared nanopores rectify ion current such that in symmetric KCl conditions on both sides of the membrane, the current migrating through the pore is higher when cations are sourced from the small opening of the pore, called the tip, compared to currents of the opposite voltage polarity.^17−19,21,36^ In all our experiments, the working electrode was placed on the side with the large opening of the pore, called the base. In this electrode configuration, negative currents are larger in magnitude than positive currents (Figure 1). Conical nanopores rectify because the pore shape in combination with the surface charge induces a voltage-dependent ionic concentration in the pore.^21,37−40^ In a negatively charged conical nanopore, negative voltages increase the concentration of both cations and anions, while positive voltages lead to the formation of a depletion zone that limits the current. The magnitude of ion current through conical nanopores and the way the current is rectified are very sensitive to changes in the pore surface charge density, pore opening diameter, and salt concentration.^25,40^ These unique characteristics make conically shaped nanopores an ideal model system to probe the effects of charge inversion on ionic transport.

To illustrate the sensitivity of the *i*–*V* curves on the surface charge, we show the *i*–*V* curves of a nanopore before and after modification with a positively charged polyelectrolyte, PAH (Figure 1C,D).^33,41^ Adsorption of the highly charged molecules led to the inversion of the *i*–*V* curve that provided evidence of the successful switch of the effective surface charge from negative to positive.^27,42^ Small standard deviations of the current before and after modification suggest that the ion current signal in KCl through these pores is stable.

The *i*–*V* curve of the as-prepared nanopore in KCl was characterized by ion current rectification, ICR(−), calculated as a ratio of negative currents at −2 V and positive currents at +2 V (Figure 1C). Recordings for the PAH-modified nanopore were described using ICR(+) calculated as a ratio of positive currents at +2 V and negative currents at −2 V (Figure 1D). Note that the PAH-modified pore rectified to a smaller degree than the as-prepared pore, which we believe could have been caused by inhomogeneous adsorption of the polyelectrolyte in the nanoconfinement of the pore.

The *i*–*V* curves before and after PAH modification will serve as a guideline in our experiments to confirm that multivalent ions indeed induced a switch of surface charge polarity from negative to positive. In the next section, we present analytical expressions that allow one to predict experimental conditions when charge inversion can be observed.

### 3.2 SCL Model of Charge Inversion Induced by Multivalent Ions

Charge inversion is an effect where counterions accumulated at a charged surface bring more charge than it is required by electroneutrality. This overcharging was postulated to result from ion–ion correlations between multivalent ions at the surface that could lead to the formation of spatial ionic ordering.^1,3^ Consequently, the position of the counterions on the surface is not random, but rather the multivalent ions create a two-dimensional SCL. For charge inversion to occur, a certain concentration threshold of multivalent ions in the bulk solution must be met, and the charge density of the Stern layer (predominantly two-dimensional layer of multivalent ions), σ_Stern_, must overcompensate the bare surface charge density, σ_b_. This concentration threshold, *c*_0_, can be calculated using the following equation:^1,4^
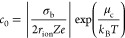
1where *r*_ion_ is the ionic radius, σ_b_ is the bare pore surface charge density, *Z* is the ion valence, *e* is the elementary charge of an electron, *k*_B_ is the Boltzmann constant, *T* is the temperature, and μ_c_ is the chemical potential due to correlation effects that is equal to
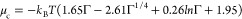
2

The parameter Γ, also commonly referred to as the Coulomb coupling constant, is the interaction parameter between the multivalent ions in the Stern layer and is defined as
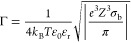
3where ε_r_ is the dielectric constant and ε_0_ is the permittivity of free space.^3^ Note that the SCL theory holds for Γ ≫ 1, which is typically fulfilled for Z ≥ 2, while the ion correlation effect is negligible for Γ ≪ 1.^3^ Assuming that σ_b_ = |−80| mC/m^2^ and ε_r_ = 80, Γ equals to 0.9 and 4.6 for KCl and tris(ethylenediamine)chromium(III) sulfate, respectively. The resulting μ_c_ are −0.86 *k*_B_*T* and −6.2 *k*_B_*T*, respectively. If *c*_bulk_ < *c*_0_, the effective surface charge of the pore walls will remain negative because the density of adsorbed ions in the Stern layer, |σ_Stern_|, is lower than the bare surface charge density, |σ_b_|. When *c*_bulk_ = *c*_0_, the effective surface charge is neutralized and |σ_Stern_| = |σ_b_|. Eventually, increasing the bulk concentration further so that *c*_bulk_ > *c*_0_ leads to charge inversion such that the density of adsorbed multivalent ions |σ_Stern_| exceeds |σ_b_|, and the effective surface charge of the pore walls switches sign.

Utilizing eq 1, we predict the threshold concentration of tris(ethylenediamine)chromium(III) sulfate (Cr^3+^) needed to induce charge inversion (*c*_0_) to be 0.6 mM, again assuming that σ_b_ is equal to |−80| mC/m^2^ and 2*r*_ion_ = 0.89 nm. The diameter 2*r*_ion_ was assumed equal to the diameter of the previously reported trivalent cobalt(III) sepulchrate,^7,20^ as confirmed by an estimate using the Cambridge Crystallographic Data Centre. Below, we show experiments that test the prediction of the model using nanopores with different opening diameters.

### 3.3 Ionic Transport, Rectification, and Current Instabilities Induced by Charge Inversion

**Figure 2:**
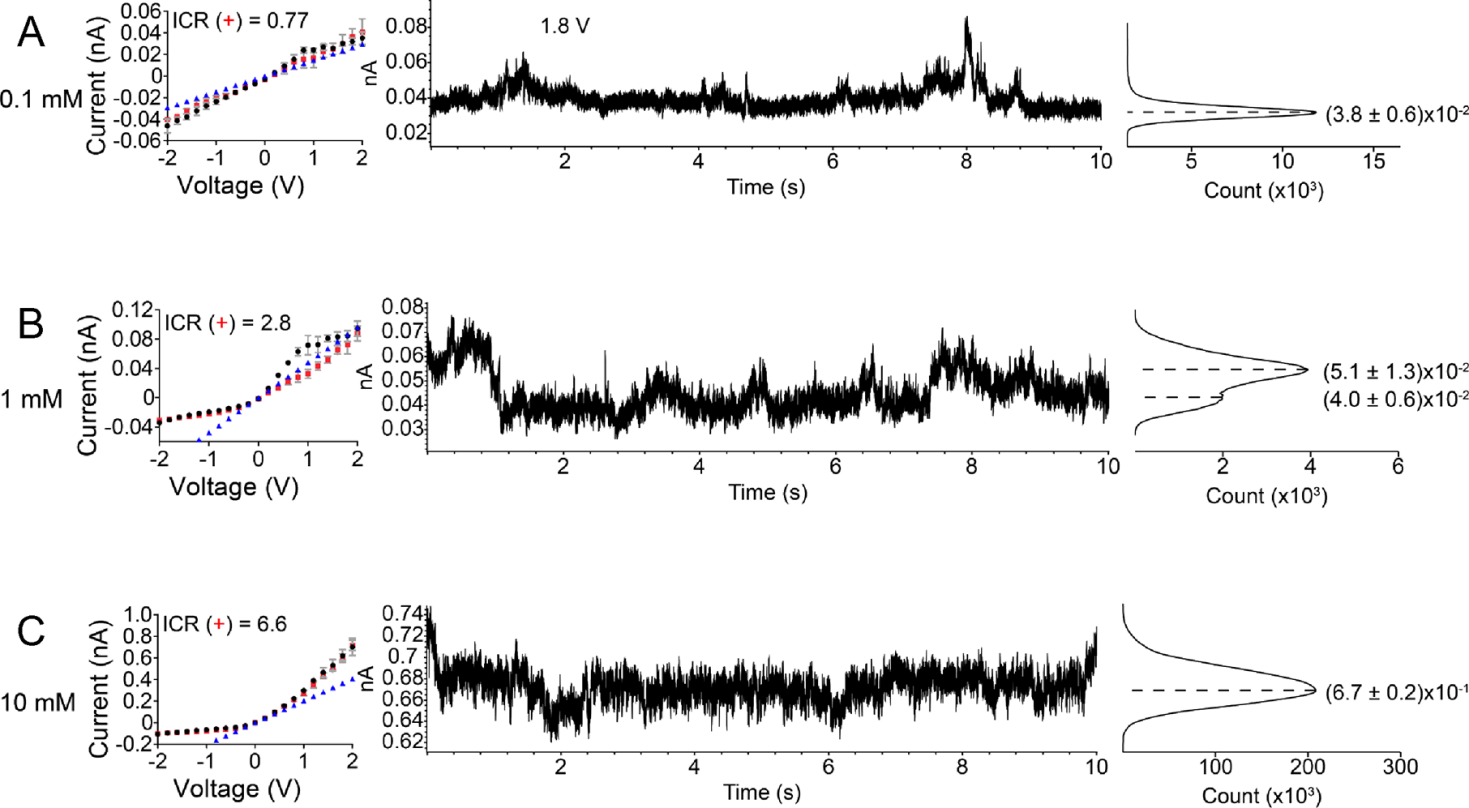
*i*–*V* curves and current time series (at 1.8 V) with histograms of a conical nanopore with a small opening of 25 nm. (A–C) The recordings were performed at pH 8 with symmetric concentrations of tris(ethylenediamine)chromium(III) on both sides of the membrane: 0.1 mM (A), 1 mM (B), and 10 mM (C). The *i*–*V* curves in the left panels were obtained by averaging 50 s long recordings of ion current in time at each voltage step. The error bars were calculated by standard deviations of ion current signals during recording. The black and red recordings indicate the forward scan (from −2 to +2 V, 200 mV steps) and the reverse scan (from +2 to −2 V, 200 mV steps), respectively. The currents in blue correspond to calculated values, assuming that the pore is uncharged and filled with the bulk solution of 0.1 mM (A), 1 mM (B), and 10 mM (C) Cr^3+^. ICR(+) is a ratio of currents at +2 and −2 V. Examples of time series of ion current are shown in the middle. Histograms of the ion current values are shown to the right with positions of the peaks and standard deviations obtained by fitting with Gaussian distribution. The large opening of this pore was 680 nm.

**Figure 3:**
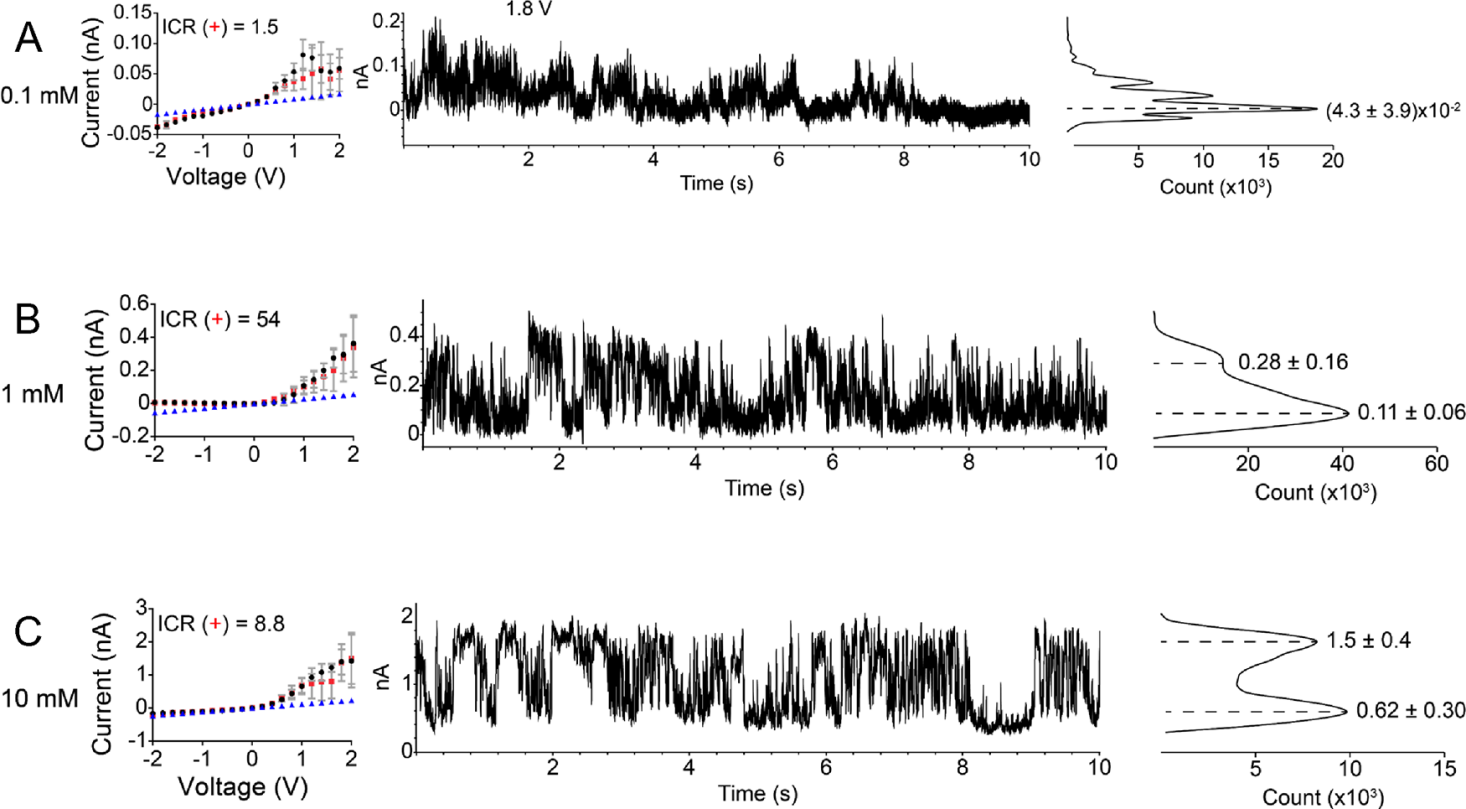
(A–C) *i*–*V* curves and current time series (1.8 V) with histograms of a conical nanopore with a small opening of 10 nm. The recordings were performed in three concentrations of tris(ethylenediamine)chromium(III): 0.1 mM (A), 1 mM (B), and 10 mM (C). The *i*–*V* curves shown in the left panels were obtained by averaging 50 s long recordings of ion current in time at each voltage step. The error bars were calculated by standard deviations of ion current signals during recording. The forward and reverse scans are shown in black and red, respectively. The currents in blue correspond to calculated values, assuming that the pore is uncharged and filled with the bulk solution of 0.1 mM (A), 1 mM (B), and 10 mM (C) Cr^3+^. ICR(+) is a ratio of currents at +2 and −2 V. Histograms of 10 s long ion current series in the middle column are shown to the right. The histograms contain positions and standard deviations of the major peaks obtained by fitting with Gaussian distribution. The base opening of this pore was 1 μm.

**Figure 4:**
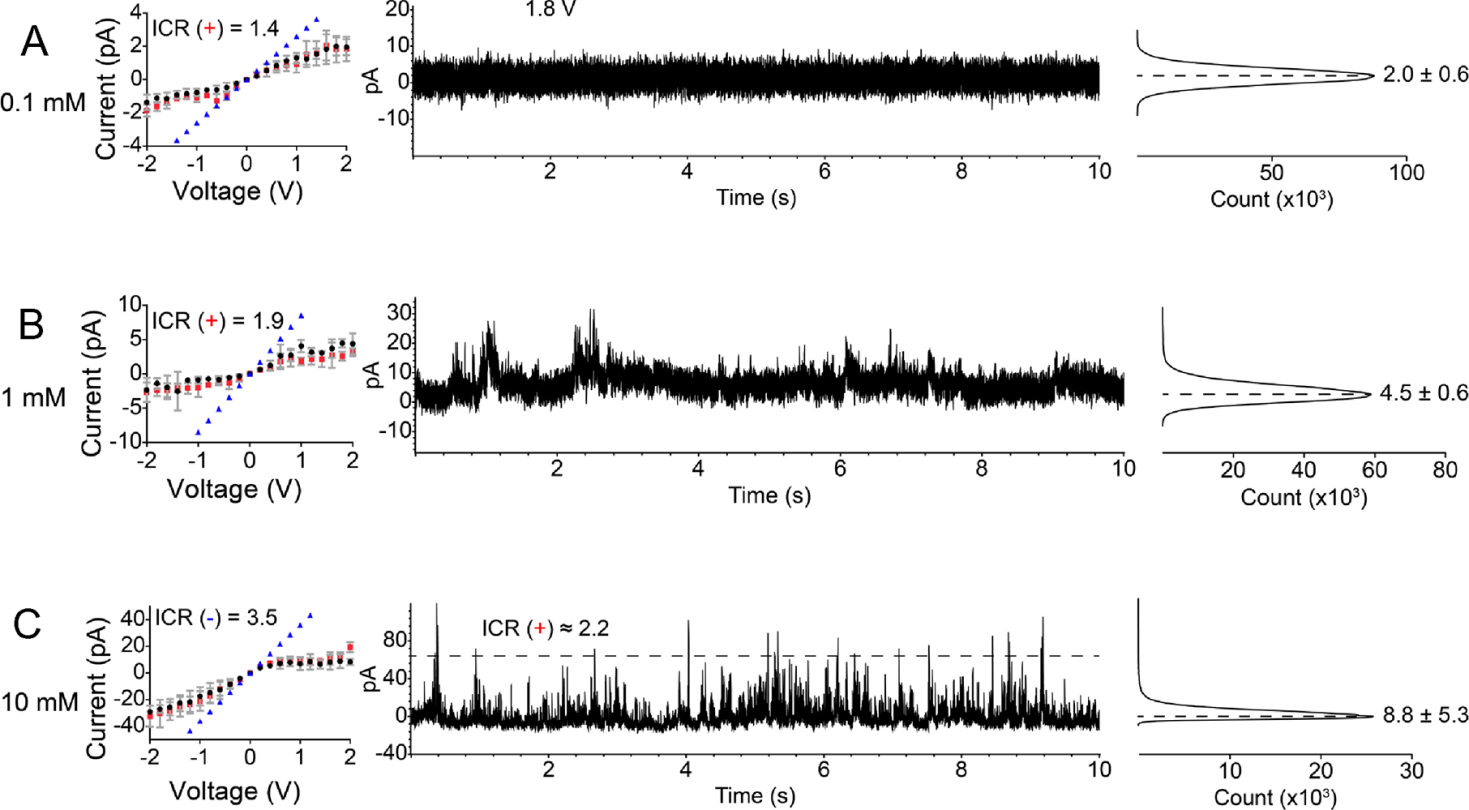
(A–C) *i*–*V* curves and current time series (1.8 V) with histograms of a conical nanopore with a tip opening of 4 nm. The recordings were performed in three concentrations of tris(ethylenediamine)chromium(III): 0.1 mM (A), 1 mM (B), and 10 mM (C). *i*–*V* curves are shown in the left panels. The black and red recordings show the forward and reverse scans, respectively. The currents in blue correspond to values calculated, assuming that the pore is uncharged and filled with the bulk solution of 0.1 mM (A), 1 mM (B), and 10 mM (C) Cr^3+^. Histograms of 10 s long ion current series in the middle column are shown to the right. The histograms contain positions and standard deviations of the major peaks obtained by fitting with Gaussian distribution. In (C), ICR(−) was calculated based on average values shown in *i*–*V* curves, while ICR(+) was found based on the transient openings of the current seen in current time series and current at −1.8 V. The base opening of this pore was 720 nm.

Four nanopores with opening diameters between 25 and 4 nm have been probed in 0.1, 1, and 10 mM tris(ethylenediamine)chromium(III) sulfate in the order from the lowest to the highest concentration. We hypothesize that introduction of the bulky multivalent ions will influence ion current by two opposite effects. First, the correlated chromium ions can lead to steric hindrance such that their layer effectively diminishes the tip diameter of the pore. The second effect is the increase of the local positive charge as more chromium ions get adsorbed, leading to the accumulation of negative sulfate ions and a higher conductance. It is noted that these two effects become more evident with the increase of Cr^3+^ bulk concentration and applied voltage. The recordings with four different pores will allow us to understand which effect dominates in pores with different opening diameters. Consequently, we will identify conditions when charge inversion can enhance or block the transport. The analysis of enhanced or blocked transport will be facilitated by comparing experimental recordings of ion current for each pore with ion current values estimated, assuming that the nanopore was uncharged and thus filled with bulk solutions (blue triangles in Figures 2–4). If Cr^3+^ ions do exert a significant steric effect, the measured currents will be smaller than the values obtained based on bulk conductivity (Table S1). Transport that is enhanced due to the presence of surface charge will be observed as currents that are higher than values calculated for an uncharged pore.

#### 3.3.1 Transport Properties of the 25 nm in Diameter Conical Nanopore

Figure 2 shows the *i*–*V* curves and current time series of a conically shaped nanopore with a 25 nm tip opening diameter recorded in various concentrations of Cr^3+^. All *i*–*V* curves were obtained by averaging the current time signals. At 0.1 mM Cr^3+^, the nanopore behaved like an ohmic resistor exhibiting a nearly linear *i*–*V* curve (Figure 2A). This recording suggests that the effective surface charge was neutralized and the bulk concentration of Cr^3+^ was insufficient for charge inversion to occur. This observation is in accordance with eq 1 that predicts 0.6 mM Cr^3+^ to be the threshold concentration required for charge inversion. It is noted though that at 0.1 mM, the pore conductance was very similar to the value calculated based on the bulk conductivity of the Cr^3+^ salt (Table S1 in the Supporting Information) and the pore geometry (blue triangles in Figure 2A). This result indicates that the bulky chromium ions did not induce any significant steric effects on the measured current in this pore, although the pore did exhibit brief bursts of higher current at positive voltages.

When the bulk concentration of Cr^3+^ reached 1 mM, the directionality of the *i*–*V* curve was inverted (Figure 2B), compared to KCl in a negatively charged as-prepared pore (Figure 1C), suggesting that the pore walls became effectively positively charged, and the pore was then anion selective. The existence of the effective positive surface charge at this concentration agrees with the theoretical prediction based on eq 1 that charge inversion is expected to occur when the bulk concentration of Cr^3+^ exceeds 0.6 mM. The *i*–*V* curve also had the same direction of rectification as the recording obtained using symmetric concentrations of KCl for a positively charged pore modified with PAH (Figure 1D). These findings are in agreement with previous experimental results showing the switching of surface charge polarity (from negative to positive) of silicon dioxide nanochannels^7,10^ and PET nanopores using other trivalent cations, CoSep^9^ and La^3+^,^14^ as well as divalent ions.^9,13,14^

It is at 1 mM Cr^3+^, however, that we started to see some additional transport properties, such as hysteresis and more pronounced ion current instabilities, effects that were not present in KCl solutions (Figure S1). As the voltage was scanned in the forward direction, from −2 to +2 V, the positive currents were larger in magnitude than when the voltage was scanned in the opposite direction from +2 to −2 V. The ion currents at high positive voltages exhibited fluctuations in time that were absent in current signals at negative voltages (Figure S2). It is also noted that when the Cr^3+^ concentration increased from 0.1 to 1 mM, the current at positive voltages increased by a factor of two, and the currents at negative voltages remained unchanged. Although the pore resistance for positive voltages was nonlinear for the forward direction, the average currents stayed close to the values calculated based on the bulk conductivity (blue triangles in Figure 2B). As the concentration of Cr^3+^ was increased to 10 mM (Figure 2C), the *i*–*V* curve became more asymmetric (see values of ICR(+) in Figure 2B,C), and the positive ion currents again increased nonlinearly with the increasing voltage for both the forward and reverse scans. At this concentration, the positive currents were much higher than what was calculated based on the bulk conductivity. This suggests that the higher concentration of chromium ions led to a higher effective positive surface charge density on the pore walls, an accumulation of mobile SO_4_^2–^ ions, and an enhanced ion conductance. The ion current instabilities remained at a similar level as in 1 mM Cr^3+^.

Existence of ICR in a 25 nm pore in the presence of multivalent ions might seem surprising because the electrical double layer, calculated from the classical Gouy–Chapman model, is significantly smaller than the pore opening radius.^43^ ICR in conical nanopores requires the pores to be partially permselective such that the applied voltage of one polarity causes enhancement of ion concentration and depletion for the opposite polarity.^19,21^ It was shown however that when charge inversion occurs, the surface potential is primarily dominated by the adsorbed multivalent ions that cause charge inversion and not by the diffuse layer.^44,45^ Moreover, the surface potential was shown to increase with the increase of the counterion diameter, an effect that was especially significant for multivalent counterions.^44,45^ Indeed, 20 nm high nanofluidic channels were found permselective in conditions when charge inversion occurred even in 10 mM MgCl_2_.^10^ Note also that the Gouy–Chapman model cannot be applied in our system because it treats ions as point charges.^43^ Consequently, even the 25 nm in diameter nanopore in Cr^3+^ is expected to be partly permselective and capable of rectification. It is also important to note that the structure of the solid/liquid interface in the presence of multivalent ions can show oscillations of cation–anion densities indicative of a layered structure of ions.^46^ In our case of negatively charged pore walls, there would be the first peak corresponding to the enhanced concentration of Cr^3+^ at the surface, followed by another peak of enhanced concentration of divalent sulfate ions, and possibly another peak of Cr^3+^. This layered structure shown before for ionic liquids^47^ leads to additional extension of the range of electrostatic interactions into the solution.

The 25 nm nanopore was large enough that the finite size of ions did not influence the average magnitude of ion current seen in *i*–*V* curves. The increased ion current instabilities seen in ion current time series at higher positive voltages might, however, stem from the steric effects of the accumulated ions, as will be discussed in more detail below. Observation of possible steric obstruction of a 25 nm nanopore suggests that the effect was not caused by individual Cr^3+^ ions, which would induce too small changes to be observed with a pore of this size. Rather, we suspect the current instabilities resulted from a collective effect of multiple ions, especially as they were seen in higher concentrations of Cr^3+^ and for the voltage polarity that enhanced ionic concentrations in the pore.

#### 3.3.2 Ion Current Enhancement and Instabilities in the 10 nm in Diameter Pore

Figure 3 shows the *i*–*V* curves and examples of the ion current in time signals for a conical nanopore that had a 10 nm tip opening diameter. This pore exhibited charge inversion at the lowest probed concentration, 0.1 mM Cr^3+^, a concentration that is six times lower than the value predicted by eq 1 (Figure 3A). This observation suggests that due to the presence of surface charges, ionic concentrations in the pore were higher than in the bulk.

As the concentration of Cr^3+^ increased to 1 and 10 mM, the ICR became more pronounced (Figure 3B,C). Interestingly, the average currents for the positive voltages at all the examined concentrations were greater than the values calculated based on bulk conductivity, suggesting that the enhanced local concentration of ions, and not the steric effects, dominated the transport properties of this nanopore. It is known that smaller conical nanopores induce a greater enhancement of the local ionic concentration for one voltage polarity than larger pores.^40^ Consequently, the 10 nm nanopore rectified stronger than the 25 nm nanopore shown in Figure 2.

The 10 nm pore, however, exhibited significant fluctuations of the ion current in time. At positive voltages, the pore switched between being closed to a finite set of open state values (Figure 3A–C). We postulate that the ion current fluctuations originated from the voltage-dependent accumulation of chromium ions in the volume of the pore and the voltage-dependent formation of aggregates of correlated ions. This hypothesis is supported by the presence of ion current fluctuations only for the voltage polarity that enhances ionic concentrations in the pore. Ion current signals at negative voltages were quite stable (Figure S2).

What could be however the chemical basis for the accumulation and release of correlated ions in the experiments here? This question is important because current instabilities were not observed in our earlier experiments with trivalent cobalt, CoSep chloride.^9^ A possible answer to the question can be gleaned from experimental and modeling studies of cation and anion distribution at a mica surface at conditions when charge inversion could occur.^48^ High-resolution X-ray reflectivity revealed the influence of anions on the overall arrangement of ions at the solid/liquid interface such that larger anions had a larger tendency to accumulate next to the counterions’ peak at the surface. Note that the study with mica^48^ reported charge inversion with monovalent rubidium cations, which could have been facilitated by the crystal structure of the substrate. In experiments reported in this manuscript, we used a sulfate anion that is significantly larger than previously used chloride in CoSepCl_3_.^9^ Thus, accumulation of sulfate could then lead to the formation of small aggregates of the salt at the surface, whose formation and dissolution would be responsible for ion current fluctuations. Another explanation for the ion current instabilities could be provided, taking into account so-called electrostatic complexes, i.e., tiny aggregates consisting of counterions, surface groups, and water.^49^ Formation of such electrostatic complexes was observed in a system of 1,2-dimyristoyl-*sn*-glycero-3-phosphate (DMPA) in contact with BaCl_2_.^49,50^ These complexes were postulated to induce surface charge inhomogeneities, and we hypothesize that they could be removed by the application of electric field, leading to current instabilities. Surface charge inhomogeneities and formation of the aggregates could be in addition facilitated by the roughness of polymer pore walls.^51^

Using the aggregate hypothesis, the ion current fluctuations and hysteresis can be explained in the following way. An initial increase of positive voltage in the forward bias leads to an increase of the number of chromium ions in the pore and increased density of correlated chromium on the pore walls that enhance the concentration of the mobile sulfate ions and ion current. As the density of Cr^3+^ ions at the surface reaches a threshold,^52^ unstable aggregates are formed, leading to pore blockage and consequently current decrease. Since the nanopore contains now a constriction (or several constrictions if more aggregates are formed), the electric field will be focused on the aggregates that create the highest resistance region in the pore. The focused electric field can facilitate the aggregate dissolution or pushing through the pore, which would lead to current increase.^38,53^ When the voltage is scanned in the reverse bias, the aggregates are not cleared immediately; consequently, this branch of *i*–*V* curves has lower currents than in the forward bias. As mentioned above, however, for this nanopore, the averaged in time currents for positive voltages were still significantly higher than values calculated for an uncharged nanopore.

Note that ion current instabilities and current hysteresis were observed before in larger conical nanopores with an opening diameter of a few tens of nanometers in contact with LaCl_3_, CaCl_2_, and MgCl_2_.^14^ Due to the larger pore diameter, the fluctuations occurred between two finite conductance states. We believe that the hypothesis we propose here could explain charge inversion-induced instabilities in pores of any size. In the case of the transport through the larger conical pores,^14^ the instabilities could be in addition affected by the formation of weakly soluble hydroxides of multivalent cations.^38^

#### 3.3.3 Ion Current Blockage in 6 and 4 nm in Diameter Pores

The steric effects that caused fluctuations of ion current in the 25 and 10 nm in diameter pores became more significant in a 6 nm pore (Figure S3) and dominated the transport of a 4 nm pore (Figure 4), where the diameter of Cr^3+^ was half the radius of the pore, enabling significant steric blockage. For the smallest pore studied, ion current in 0.1 mM Cr^3+^ had nearly zero conductance, suggesting that the trivalent ions and their aggregates completely shut off the transport (Figure 4A). The nanopore continued to be mostly closed in 1 mM Cr^3+^ where only occasional bursts of finite conductance were observed (Figure 4B). Finally, as the concentration of chromium ions reached 10 mM, average negative currents significantly increased such that the nanopore rectified as if it was still negatively charged. The shape of the *i*–*V* curves at 10 mM Cr^3+^ is the most surprising observation for this pore, suggesting that even the formation of the correlated liquid crystal structure could be hindered in this pore (Figure 4C). The time series of ion current at both voltages, however, revealed that there were a few brief instances of time when positive currents were higher than negative currents, as expected from charge inversion. Due to the small opening of the nanopore, the aggregates of ions could lead to nearly complete pore closure; however, when open, the pore still exhibited transport properties indicative of its effective positive charge. This study underscores the importance of probing signals of ion current in time since the averaged *i*–*V* curves do not reflect all important processes that dynamically occur in nanopores. Positive currents are suppressed nearly completely in the smallest nanopore because the aggregates occupy the pore volume for prolonged periods of time blocking the current (Figure 4C). Even small aggregates will block the pore due to the comparable size of the Cr^3+^ ions and the pore radius.

When comparing the magnitudes of ion current in −2 V and in open bursts at +2 V (Figure 4C), we realized that the rectification was weaker than seen in the 25 and 10 nm pores in the same Cr^3+^ concentration. This observation indicates that the effective charge density induced by charge inversion can be pore diameter dependent.

### 3.4 Continuum Modeling of Pore Diameter and Voltage-Dependent Charge Inversion

To interpret the experimental observations of charge inversion in nanopores of different opening diameters, we applied two models, referred here as Model 1 and Model 2. In Model 1, the surface charge density is described by the SCL theory,^1^ as abovementioned. In Model 2, we use the site-binding approach based on the Langmuir adsorption isotherm to determine charge density as a function of Cr^3+^ concentration (Supporting Information).^54−56^ Model 2, therefore, takes into account correlations between counterions and interfacial charges that were also found important for charge inversion.^49,57^ Each model has its advantages and disadvantages. Model 1 captures correlation between counterions and describes equilibrium state of charged surfaces placed in contact with high valence counterions. In Model 1, however, the predicted surface charge of the pore walls, σ_eff_, is determined by eq 1 and remains independent of the transmembrane potential. Constant σ_eff_ can be problematic because applied voltage is known to affect ion concentrations in a conical nanopore. Model 2, on the other hand, predicts voltage-dependent σ_eff_ through voltage-dependent ionic concentrations, but it neglects the electrostatic correlation between the multivalent ions accumulated at the surface. In addition, equilibrium constants in Model 2 are treated as fitting parameters and simple adsorption mechanism may not estimate the local σ_eff_ correctly. Since the pore size considered experimentally was between 25 and ∼4 nm for the tip region, the steric effect due to the finite ionic size was considered in both models.^52^ Here, we assumed that Cr^3+^ and SO_4_^2–^ ions have equal effective ion sizes of 0.89 nm in diameter reported for CoSep.^4,20^ Ion current was modeled based on coupled Poisson–Nernst–Planck (PNP) and Navier–Stokes (NS) equations (see the Supporting Information file for details).

**Figure 5:**
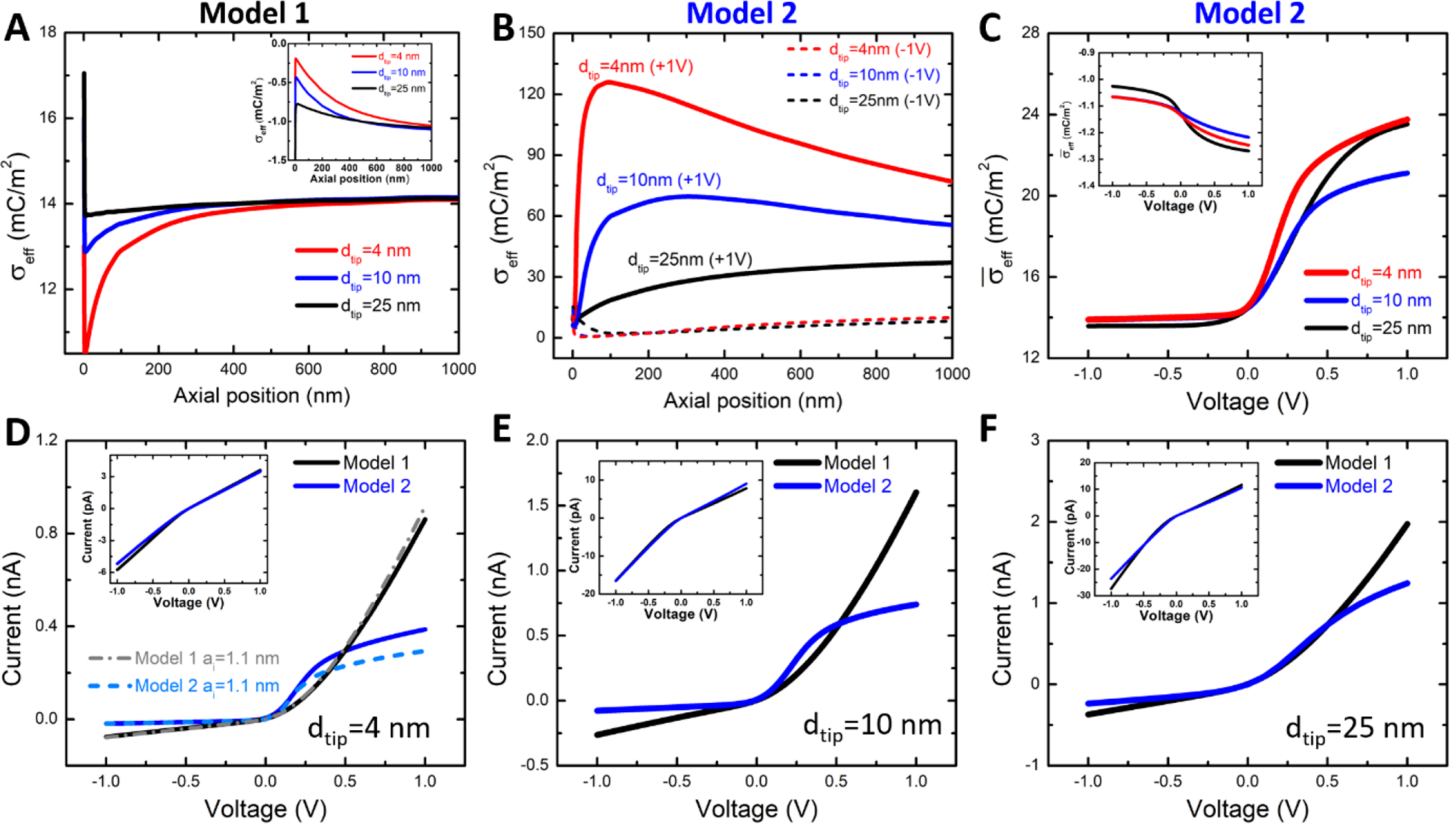
Numerical modeling of charge inversion using the SCL theory (Model 1) and the site-binding model (Model 2), assuming that the effective diameter of cations and anions equals to 0.89 nm. (A) Axial variation of σ_eff_ along the pore axis for 0.1 mM (inset) and 10 mM Cr^3+^ at three different tip openings, as predicted by Model 1. (B) Axial variation of σ_eff_ in 10 mM Cr^3+^ for 4, 10, and 25 nm in diameter nanopores, as predicted by Model 2. (C) Average effective surface charge density from Model 2 for different pores and voltages at 0.1 mM (inset) and 10 mM Cr^3+^. (D–F) Simulated *i*–*V* curves at 0.1 mM (insets) and 10 mM Cr^3+^ for three tip opening diameters of (D) 4 nm, (E) 10 nm, and (F) 25 nm from Model 1 (black lines) and Model 2 (blue lines) using coupled PNP–NS equations. The dashed lines in (D) show the *i*–*V* curves simulated for the same pore geometry but larger ions; the diameter of both cations and anions was assumed 1.1 nm. In (A) and (B), the axial position of 0 denotes the location of tip opening. Figure S6 shows a larger portion of the simulated system for Figure 5A,B. The base openings are similar to the values reported for the pores shown in Figures 2–4.

Figure 5 shows the σ_eff_ obtained from Model 1 and Model 2 as well as the simulated *i*–*V* curves for various combinations of pore geometry and bulk concentration. As expected, σ_eff_ is negative at 0.1 mM Cr^3+^ and turns to positive values at 10 mM Cr^3+^ for all pores. Figure 5A and Figure S4 show in detail the steric effects of Cr^3+^ on the effective surface charge density, as predicted by Model 1. The steric effects are especially visible at a more confined space, higher salt concentrations, and higher surface charge densities (Figure S4). As an example, in 10 mM Cr^3+^, Model 1 predicts the surface charge at the tip to be nearly 14 mC/m^2^ in the 25 nm in diameter pore but only 11 mC/m^2^ in the 4 nm pore (Figure 5A). Interestingly, the surface charge density is dependent on the pore diameter even in 0.1 mM Cr^3+^ and thus in a concentration that is below the threshold concentration for charge inversion to occur (inset to Figure 5A). Here, as the diameter of the pore decreases, the surface charge becomes less negative. Since the pores used here are conically shaped, the effective surface charge density is axially inhomogeneous, especially within the first ∼600 nm from the tip. Figure 5A shows the effective surface charge within the first 1 μm long segment from the tip opening where the changes are most pronounced. The modeled pore was 11 μm long, and the surface charge along the whole length of the pore is shown in Figure S6A.

Figure 5B shows σ_eff_ calculated with Model 2 for three nanopores with different opening diameters (25, 10, and 4 nm) at −1 and +1 V in the first 1 μm long region at the tip, where the changes along the pore axis are again the strongest. Note that σ_eff_ along the whole pore length is shown in Figure S6B. Here, σ_eff_ is a function of not only the pore opening diameter but also of the applied voltage. In 10 mM Cr^3+^, the pore wall carries a higher positive charge density at positive voltages than at negative voltages. Note that the inhomogeneous surface charge occurs over a significantly larger portion of the pore walls, compared to predictions from Model 1 (Figure 5A and Figure S6). The polarity-dependent surface charge results from the accumulation/depletion of cations that occurs in the pore near the tip opening at positive/negative voltages, thereby increasing/decreasing the magnitude of σ_eff_. Consequently, σ_eff_ exhibits a local maximum (minimum) at +1 V (−1 V) near the tip opening. Since the local ion concentrations are higher in narrower pores, σ_eff_ for positive voltages is the highest at the tip for the 4 nm in diameter nanopore.^40^ The highly enhanced σ_eff_ at +1 V can be two orders of magnitude larger than the reduced σ_eff_ at −1 V and might not be realized experimentally. This inhomogeneous and voltage-dependent σ_eff_ also supports our hypothesis of voltage-dependent accumulation/depletion of multivalent ions at the surface that could lead to ion current instabilities in time. Note that the saturated σ_eff_ at +1 V near the tip opening indicates the theoretical limit of the Langmuir isotherm.

To facilitate comparison of the effective charge between different pores and voltages, we also calculated the average surface charge, σ̅_eff_, as predicted by Model 2. Figure 5C shows the σ̅_eff_ obtained by averaging the surface charge over the entire length of the nanopore, 11 μm. Interestingly, at 10 mM Cr^3+^, σ̅_eff_ (*d*_tip_ = 4 nm) is larger than σ̅_eff_(*d*_tip_ = 25 nm) at intermediate positive voltages (e.g., V < ∼+0.7 V), but when the voltage further increases, σ̅_eff_(*d*_tip_ = 4 nm) tends to level off and becomes comparable to σ̅_eff_(*d*_tip_ = 25 nm). This can be explained by the steric effect of Cr^3+^ at a nanoconfinement that becomes stronger at higher salt concentrations. In this case, the voltage-driven increase of ion concentration near the tip opening is weakened. In 0.1 mM Cr^3^, Model 2 predicts that the pore walls are still negatively charged but with voltage-dependent σ̅_eff_(inset to Figure 5C). Finally, the dependence of the surface charge density on pore diameter is also evident from the modeling based on Model 2 performed at 0 V (Figure S5). Similar to the results obtained with Model 1, the surface charge density at the tip is the lowest, which is especially evident for the 4 nm pore.

Figure 5D–F shows the *i*–*V* curves for various concentrations and tip openings found based on Model 1 (black curves) and Model 2 (blue curves). In general, the rectification exhibits opposite directions in 0.1 and 10 mM Cr^3+^, which points to the charge inversion in the higher concentration. For positive voltages below 0.5 V, ion currents calculated based on Model 2 are higher than the predicted currents from Model 1 due to voltage-induced enhancement of ions in the pore. As the magnitude of voltage increases, ion currents from Model 2 begin to plateau due to the steric effects of the ions that limit a further increase of ionic concentration in the pore (blue and orange curves in Figure S7).

We also considered the effect of convection on overall ion transport. Figure S7 shows the *i*–*V* curves calculated based on Models 1 and 2 using coupled PNP–NS and only PNP equations. Model 1 was found insensitive to the presence of convection, while Model 2 predicts significantly lower currents when NS equations are included. The larger influence of convection in Model 2 is due to the higher effective surface charge density predicted by the model. Convection also leads to lowering of ionic concentrations in the pore (Figure S8A). Note that including convection does not change the direction of rectification seen in *i*–*V* curves. We also considered more complex properties of the convective flow at the pore entrance that could contribute to the current. Figure S8B shows a velocity profile along the pore axis at 1 V that revealed a reversal of the flow velocity at the pore entrance, however, with the magnitude that was much smaller than the velocity in the pore. Since the velocity reversal was confined to the region outside the pore and the velocity magnitude was low, we believe that this local velocity profile did not significantly influence the temporal characteristics of ion current.

The inverted rectification at 10 mM and *d*_tip_ = 4 nm (Figure 4C) is not reproduced by Model 1 or Model 2. This is expected because the models do not capture ion current instabilities. Both models however predicted that the pore walls will still be effectively positively charged, in which the experimental data revealed as high amplitude bursts of current at positive voltages.

The 4 nm nanopore also served as a model system to understand the extent that the steric effect can influence *i*–*V* curves, as predicted by Model 1 and Model 2. Figure 5D shows two additional *i*–*V* curves obtained for the same pore geometry but with the diameter of both cations and anions equal to 1.1 nm versus 0.89 nm used in all other simulations. Interestingly, the currents predicted by Model 1 were nearly independent of the ion size. Model 2, on the other hand, predicted even more suppressed positive currents, which could stem from diminished concentration of ions in the pore.

We would also like to note that the early onset of charge inversion in the 10 nm pore in 0.1 mM Cr^3+^ has not been captured by the models; however, the less negative surface charge at the tip of narrow pores in 0.1 mM Cr^3+^ could contribute to changes in the *i*–*V* curves. We believe that a more complete model would have to be designed to include a possible instability of the correlated liquid and time-dependent surface charge density. Building such a model is beyond the scope of this study.

In summary, each of the models captured a subset of our experimental findings. Model 1 described the physics of charge inversion based on ion correlations and SCL but was not able to describe the possibility of modulating the effective surface charge density by the transmembrane electric field. Model 2, on the other hand, captured the voltage dependence of the local surface charge whose density could reach sufficiently high values for the accumulated/correlated counterions to become unstable. We hypothesize that the repeated accumulation and release of the accumulated ions could be responsible for ion current instabilities in time.

## 4 Conclusions

In this manuscript, we probed experimentally and by numerical modeling charge inversion in conically shaped nanopores with different opening diameters. The switch of surface charge from negative to positive can have a huge impact on separation processes based on surface charge. The SCL theory, widely used to describe charge inversion, does not describe possible modulations of the effective charge density in nanoconfinement, yet our results suggest that in sub-10 nm nanopores, charge inversion can be hindered and will not reach the full magnitude predicted by the SCL model. We have also discovered that the correlated ions can have two competing effects on ionic current in conically shaped nanopores. On one hand, an increased density of correlated ions leads to an increase in the concentration of mobile counterions (sulfate in our experiments) observed as current increase. Correlated ions, however, also diminish the effective opening of the pore, and this steric hindrance becomes especially important in sub-10 nm nanopores. In addition, our experiments revealed ion current instabilities in 10, 6, and 4 nm diameter pores that occurred at positive voltages above 1 V. The current would switch from a state with nearly no current to a set of states with finite conductance. We postulate that this dynamic behavior can be explained by unstable aggregates formed by the correlated ions.

The manuscript demonstrates an ability to modulate transport properties of nanopores using bulky multivalent ions. Nanoconfinement-modified charge inversion will be important for designing sensors of multivalent ions^15^ as well as development of separation membranes. Our results suggest that in the presence of multivalent impurities, nanoporous membranes can switch selectivity between cationic and anionic, which would have a large impact on their performance. Moreover, the density of the induced positive charge can be further modulated by the pore diameter. Our results advance understanding how ionic transport is influenced by the nanoconfinement.^58−65^

## Supporting Information Available

Additional experimental data of current–voltage curves and ion current in time as well as modeling results together with detailed information about modeling approaches, referred to as Model 1 and Model 2 (PDF)
