# Supplementary material for: Gating with Charge Inversion to Control Ionic Transport in Nanopores
Source: ACS Appl Nano Mater. 2022 Dec 1;5(12):17682–92. doi: 10.1021/acsanm.2c03573 (PMC9791654; doi:10.1021/acsanm.2c03573)
Supplement: Supplementary file 1 — an2c03573_si_001.pdf [file an2c03573_si_001.pdf]

Supporting Information

Gating with Charge Inversion to Control Ionic Transport in Nanopores

Wilfred S. Russell,[1, #] Chih-Yuan Lin,[2, #] Zuzanna S. Siwy[1,2,3,*]

[1]*Department of Chemistry, University of California, Irvine, CA 92697, USA*

[2]*Department of Physics and Astronomy, University of California, Irvine, CA 92697, USA*

[3]*Biomedical Engineering, University of California, Irvine, CA 92697, USA*

Table S1. Bulk conductivity measurements of tris(ethylenediamine)chromium(III) sulfate at select experimental concentrations.

[Tris(ethylenediamine)chromium(III) sulfate] (mM)	Conductivity (mS/m)
0.1	13 ± 0.6
1	43 ± 0.9
10	183 ± 5

* Corresponding Authors: zsiwy@uci.edu, Tel. 714-712-0027

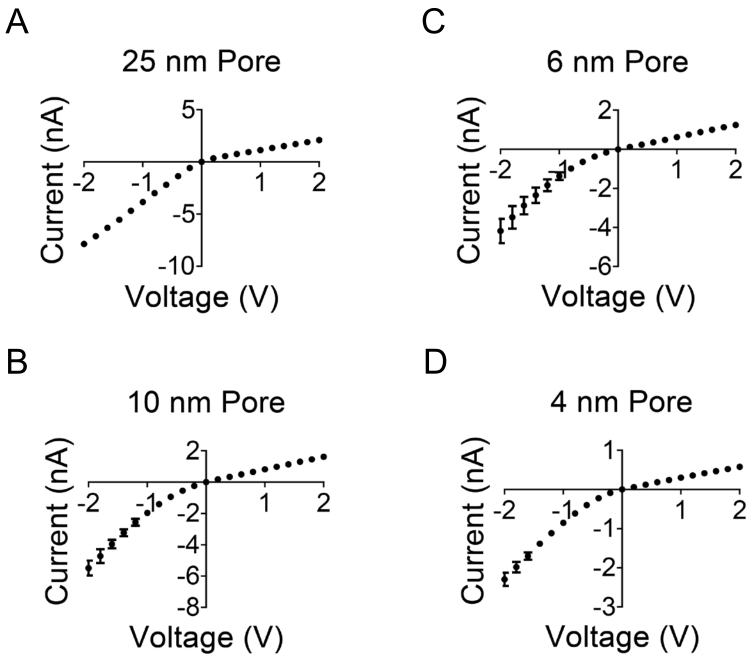

Figure S1. Current-voltage curves through as prepared nanopores recorded in 100 mM KCl. The currents shown are averages over three forward and three reverse voltage scans. Panels (A) – (D) show i-V curves for 25 nm (A), 10 nm (B), 6 nm (C), and 4 nm (D) in diameter single conical nanopores.

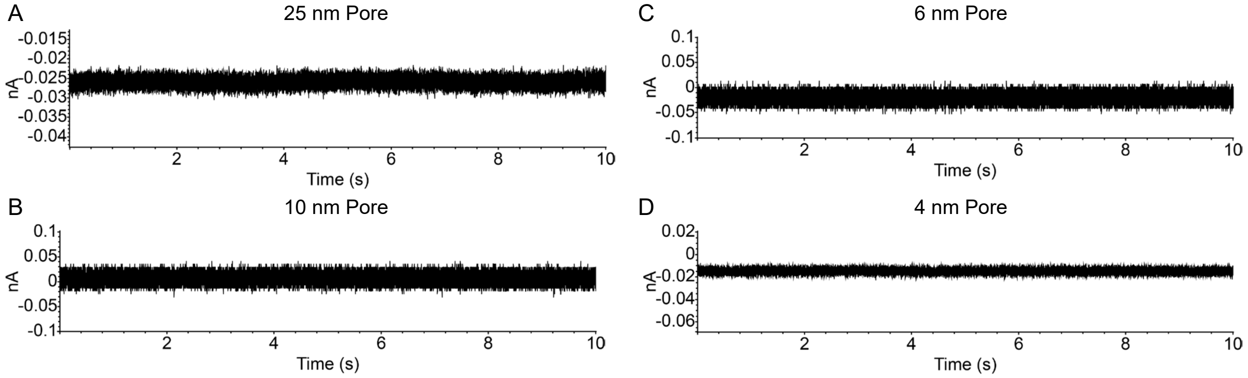

Figure S2. Ion current time series recorded in -1.8 V in the presence of 1 mM tris (ethylenediamine) chromium(III) sulfate for the four nanopores presented in Figures 2-4 in the main manuscript and Figure S3. The nanopore diameters were: (A) 25 nm, (B) 10 nm, (C) 6 nm, and (D) 4 nm. Note the lack of current instabilities in a form of switching between nearly zero conductance and finite conductance levels. The increased noise level in (B) and (C) coincides with current instabilities observed for +1.8 V shown in the main manuscript.

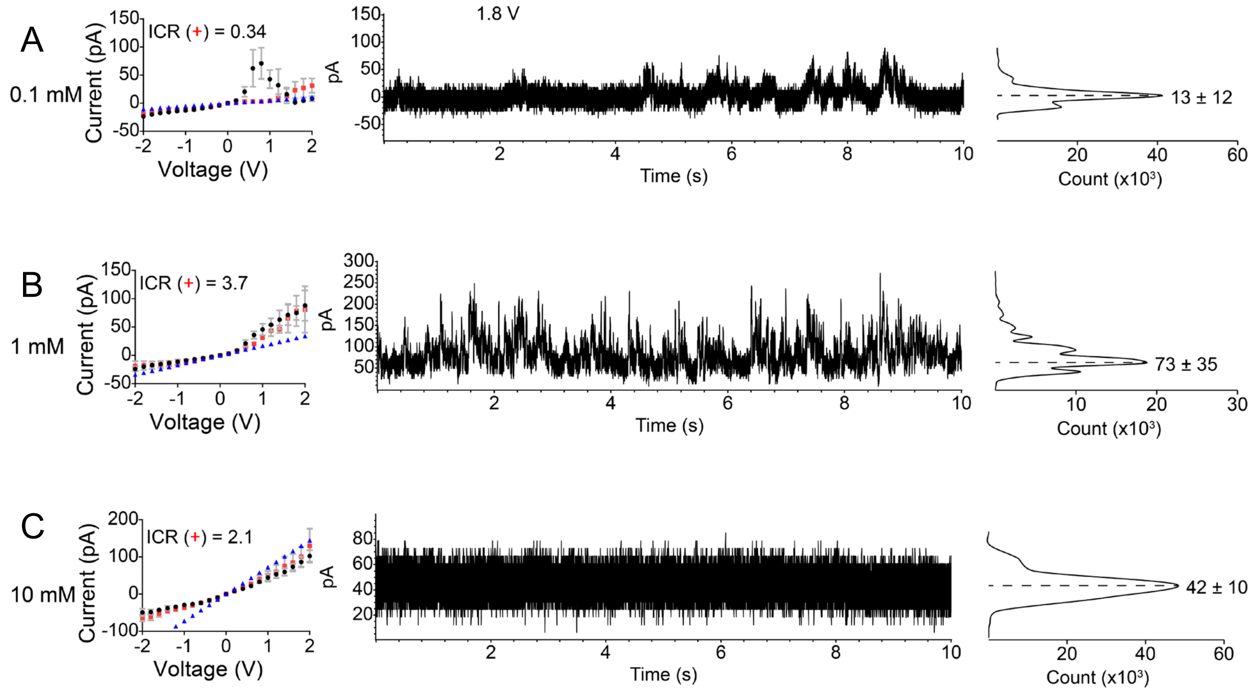

Figure S3. (A-C) Current-voltage (i-V) curves and current-time series (1.8 V) together with histograms of a conical nanopore with a small opening of 6 nm. The recordings were performed in three concentrations of tris(ethylenediamine)chromium(III): 0.1 mM (A), 1 mM (B), and 10 mM (C). i-V curves shown in the left panels were obtained by averaging 50 s long recordings of ion current in time. Error bars were calculated by standard deviations of ion current signals during recording. The recordings in black and red indicate the forward and reveres scans, respectively. The currents in blue correspond to values calculated assuming the pore is uncharged and filled with the bulk solution of 0.1 mM, 1 mM, and 10 mM Cr^{3+}, respectively. Examples of time series of ion current are shown in the middle. Histograms of the ion current values are shown to the right with positions of the peaks and standard deviations obtained by fitting with Gaussian distribution. The base opening of this pore was 1040 nm.

Modeling

The simulated system consists of two large reservoirs and an 11-μm-long conical nanopore with the tip and base opening diameters of d_{tip}, d_{base}, respectively (**Table S2**). The pore diameters used in the modeling are the same as of the pores used in the experiments: [d_{tip}, d_{base}]=[4 nm, 720 nm], [d_{tip}, d_{base}]=[10 nm, 1000 nm], and [d_{tip}, d_{base}]=[25 nm, 680 nm].

Taking into account the finite size of the ions and the electroosmotic flow, the ion transport in conical nanopores is described by the modified Poisson-Nernst-Planck (PNP) and Navier-Stokes (NS) equations[1, 2]

$$-\varepsilon_r \varepsilon_0 \nabla^2 \psi = eN_A \sum_{i=1}^{2} z_i c_i \tag{S1}$$

$$\nabla \cdot \mathbf{J}_i = \nabla \cdot \left[\mathbf{u} c_i - D_i \left(\nabla c_i + \frac{z_i e}{k_B T} c_i \nabla \psi + \frac{a_i^3 c_i \sum_{i=1}^{2} \nabla c_i}{1 - \sum_{i=1}^{2} a_i^3 c_i} \right) \right] = 0 \tag{S2}$$

$$-\nabla p + \eta \nabla^2 \mathbf{u} - eN_A \sum_{i=1}^{2} z_i c_i \nabla \psi = \mathbf{0} \tag{S3}$$

$$\nabla \cdot \mathbf{u} = 0 \tag{S4}$$

Here ψ is electric potential; c_i, D_i, z_i and a_i are the concentration, diffusivity, valence, and effective ionic size of ith ionic species, respectively; **u**, *p*, and η are the fluid velocity, hydrodynamic pressure, and dynamic fluid viscosity, respectively. The parameter a_i indicates ionic diameter: $a_i = 2r_{ion} = 0.89$ nm. The diffusion coefficients of 10.7 x 10^{-10} m^2/s were adopted for both Cr^{3+} and SO_4^{2-} ions. The boundary conditions on the pore wall include zero normal flux, nonslip, and charge density (**Table S2**). In particular, the surface charge density is determined by the interactions of multivalent counterions with a charged surface and between themselves, as described below. The coupled eqs. (S1) - (S4) and the associated boundary conditions were numerically solved with finite-element-method based software COMSOL Multiphysics.

Table S2. Simulated system and boundary conditions.

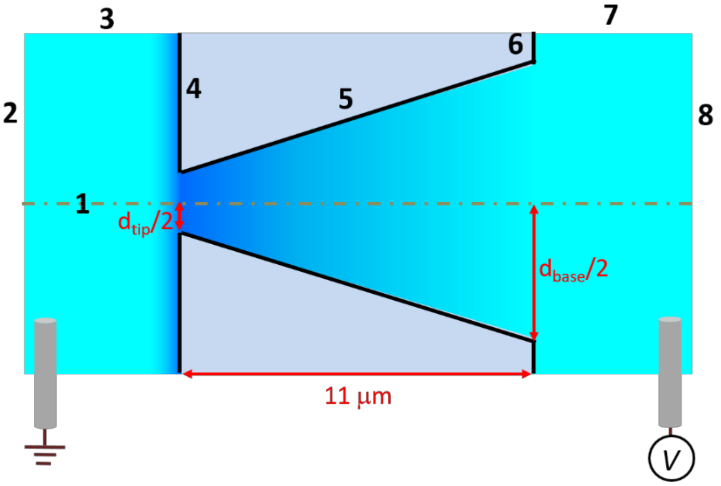

(not to scale)

<table>
<tr><th>BC</th><th>Poisson</th><th>Nernst-Planck</th><th>Navier-Stokes</th></tr>
<tr><td>1</td><td>axial symmetry</td><td>axial symmetry</td><td>axial symmetry</td></tr>
<tr><td>2</td><td>$\psi = 0$</td><td rowspan="2">$c_i = C_{i,bulk}$
(bulk concentration)</td><td rowspan="2">$p = 0$
$\mathbf{n} \cdot [\eta(\nabla \mathbf{u} + (\nabla \mathbf{u})^{\mathbf{T}})] = 0$</td></tr>
<tr><td>8</td><td>$\psi = V_{app}$</td></tr>
<tr><td>3, 7</td><td>$-\mathbf{n} \cdot \nabla \psi = 0$</td><td>$\mathbf{n} \cdot \mathbf{J}_i = 0$
(zero normal flux)</td><td>slip</td></tr>
<tr><td>4,5,6</td><td>$-\varepsilon_r \varepsilon_0 \mathbf{n} \cdot \nabla \psi = \sigma_{eff}$
(charge density)
Model 1: eq S5
Model 2: eq S7</td><td>$\mathbf{n} \cdot \mathbf{J}_i = 0$
(ion-impenetrable)</td><td>$\mathbf{u} = \mathbf{0}$
(nonslip)</td></tr>
</table>

*Upper case **T** denotes matrix transpose

Model 1

In Model 1, we introduced the strongly correlated liquid theory to describe the ion correlation effect such as electrostatic interactions between charged surface and the multivalent ions.[3-5] The effective surface charge density on the pore wall, σ_{eff}, is given by the sum of bare charge density σ_b and adsorbed Z-ions resembling Wigner crystal,[5, 6]

$$\sigma_{eff} = \sigma_b + Ze2r_{ion}C_b \exp\left(-\frac{(\mu_c + Ze\psi_s)}{k_B T}\right) \quad \text{(S5)}$$

where C_b and ψ_s are bulk concentration of Z-ion and electric potential in the Stern layer, respectively. μ_c is defined as eq. (2) in the main text. σ_b in our system is related to the dissociation of carboxyl groups from the PET surface, and equal -0.08 C/m^2; only Figure S4 shows results for σ_b = -0.16 C/m^2. The second term on the right-hand side of eq. (S5) is also referred to the charge density in the Stern layer, σ_{stern}, as described in the main text of the manuscript. In our model, σ_{stern} originates from the fact that, in equilibrium, electrical potential of Z-ions in the bulk solution (= $\mu_b^0 + k_B T \ln C_b + Ze\psi_b$) is equal to that of adsorbed Z-ions on the surface (= $\mu_s^0 + k_B T \ln(n/2r_{ion}) + Ze\psi_s$).[5, 6] n is the two-dimensional concentration of Z-ions on the surface. Note that μ_s^0 (μ_b^0) is influenced by ion correlation, hydration, and binding effects. In our case with Z = 3 and $\varepsilon_r = 80$, charge inversion is dominated by ion correlation, thereby $\mu_s^0 - \mu_b^0 \cong \mu_c$.[5] This approximation was verified in ref. 5 which compared the role of ion correlation and the role of ion binding on charge inversion. Considering Z-ion as Cr^{3+} ion, the values of Z = 3 and 2r_{ion} = 0.89 nm were adopted.

Model 2

Model 2 describes an adsorption equilibrium between the ions and the surface.[7-9] The carboxyl groups on the track-etched PET surface are negatively charged ($-COO^-$) in aqueous solutions. At pH 8, the dissociation of carboxyl groups on the nanopore surface is almost complete (~σ_b), thus the protonation/deprotonation of $-COO^-$ is negligible.

Assuming that the adsorption of Cr^{3+} ions on PET surface follows the dissociation reaction with a dissociation constant K_I,

$$-COO^- + Cr^{3+} \leftrightarrow -COO^- Cr^{3+} \tag{S6}$$

the effective surface charge density is given by:

$$\sigma_{eff} = \sigma_b \left(\frac{1 - 2K_I[Cr^{3+}]_s}{1 + 2K_I[Cr^{3+}]_s} \right) \tag{S7}$$

where $[Cr^{3+}]_s$ denotes the Cr^{3+} concentration on the pore surface. The surface charge is zero, $\sigma_{eff} = 0$, at the threshold concentration for charge-inversion, c_0 in Eq. (1), which implies that $c_0 \sim 1/2K_I$. Consequently, for simplicity, $1/K_I$ can be approximated as 1.2 mM in our modeling.

We further examine the effects of ionic size and electroconvection on the magnitude of ion current, as shown in **Figure S7**, which compares i-V curves obtained when using coupled PNP-NS as done in the main text, with i-V curves calculated using PNP only. For Model 1, the results obtained with PNP and PNP-NS equations are both qualitatively and quantitatively similar (**Figure S7A**, black and grey curves). Model 2 shows the same rectification direction in both approaches but adding Navier-Stokes significantly reduces the magnitude of positive currents, and leads to an S-shape of this branch of i-V curves (**Figures 5, S7A**, blues curves). We attribute the current reduction to: (i) lower ionic concentration inside the nanopore (**Figure S8A**) compared to values from the PNP-based model, and possibly (ii) the reversal of electroosmotic flow outside the nanopore tip opening that might limit ionic transport as well (**Figure S8B**). The latter has also been observed in other conical nanopore systems.[10, 11] Note that the flow reversal does not occur inside the nanopore, and we believe it does not influence the temporal characteristics of the measured current. Furthermore, the comparison between blue and orange curves **in Figure S7** demonstrates the importance of finite ionic size in the current suppression.

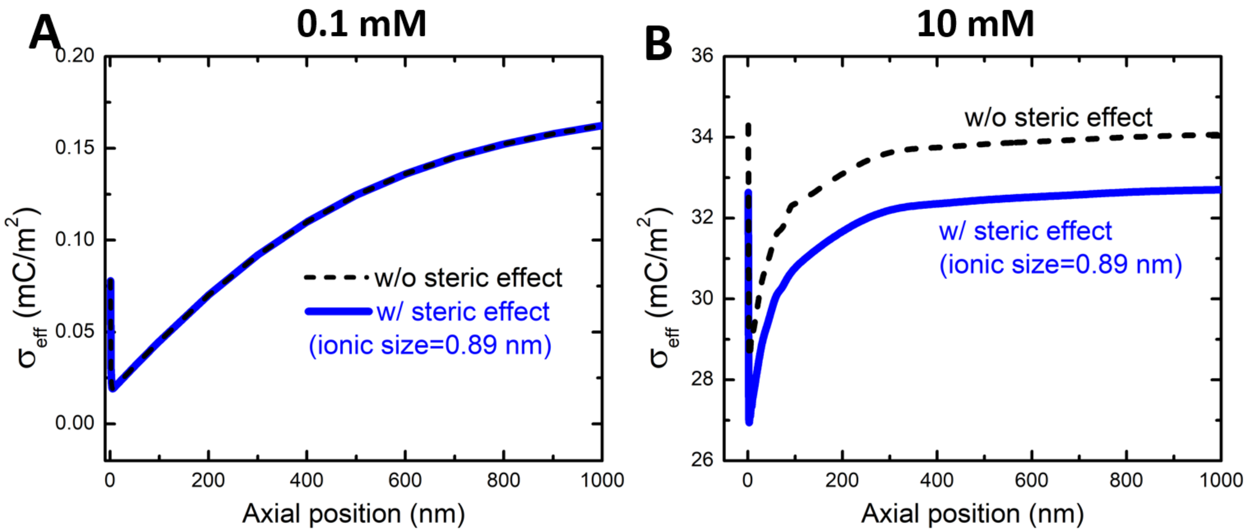

Figure S4. Effects of finite ionic size on σ_{eff}, as predicted by Model 1. Axial variation of σ_{eff} along the pore axis at 0.1 mM Cr^{3+}, (A), and 10 mM Cr^{3+}, (B), for d_{tip} = 4 nm, d_{base} = 720 nm, σ_b = -0.16 C/m^2, and 0 V. The difference in σ_{eff} between the cases with (blue curve) and without (black curve) steric effect is significant in 10 mM Cr^{3+}.

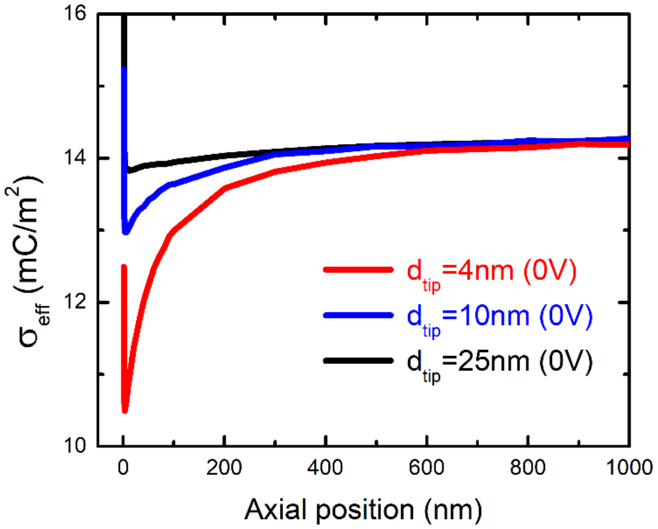

Figure S5. Axial variation of σ_{eff} along the pore axis for 10 mM Cr^{3+} at 0 V for three nanopores, as predicted by Model 2. The σ_{eff} becomes less positive as the diameter of the pore, d_{tip}, decreases.

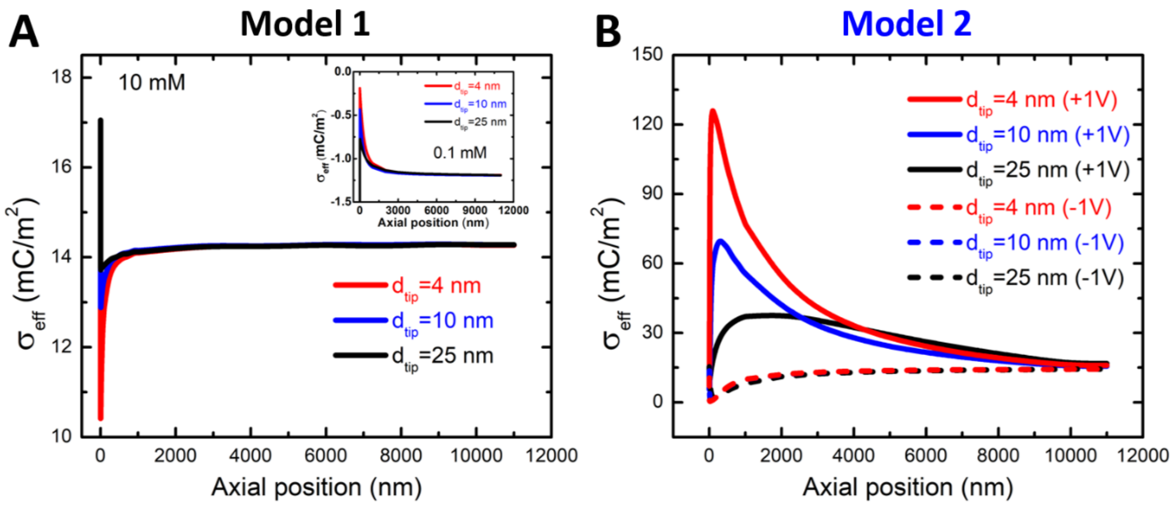

Figure S6. (A) Effective surface charge in conical nanopores with different opening diameters, as indicated in the figure, along the whole pore length of 11 µm calculated according to Model 1. Results for 10 mM and 0.1 mM (inset) Cr^{3+} are shown. Figure 5A shows a zoomed in portion between 0 (the tip) and 1 µm. (B) Effective surface charge of three conical nanopores along the pore length of 11 µm calculated according to Model 2 for 10 mM and 0.1 mM (inset) Cr^{3+}. Figure 5B shows a zoomed in portion between 0 (the tip) and 1 µm. This Figure is complementary to Figure 5A and Figure 5B in the main text.

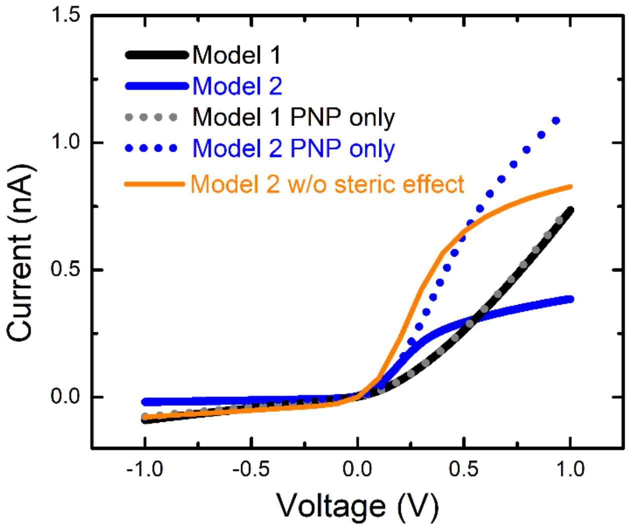

Figure S7. Effects of convection and finite ionic size on the magnitude of ion currents. Simulated i-V curves at 10 mM Cr^{3+} for a nanopore with the tip opening diameter of 4 nm. Black and blue curves are the same results as shown in Figure 5D and were obtained based on coupled PNP-NS equations. Grey-dotted and blue-dotted lines present results from Model 1, and Model 2, respectively without convection i.e. the convection term of Eq. S2 and Navier-Stokes equations were neglected in the simulation. Orange solid-curve represents the result of Model 2 without considering the steric effect (i.e., ionic size a_i =0 in eq. (S2)).

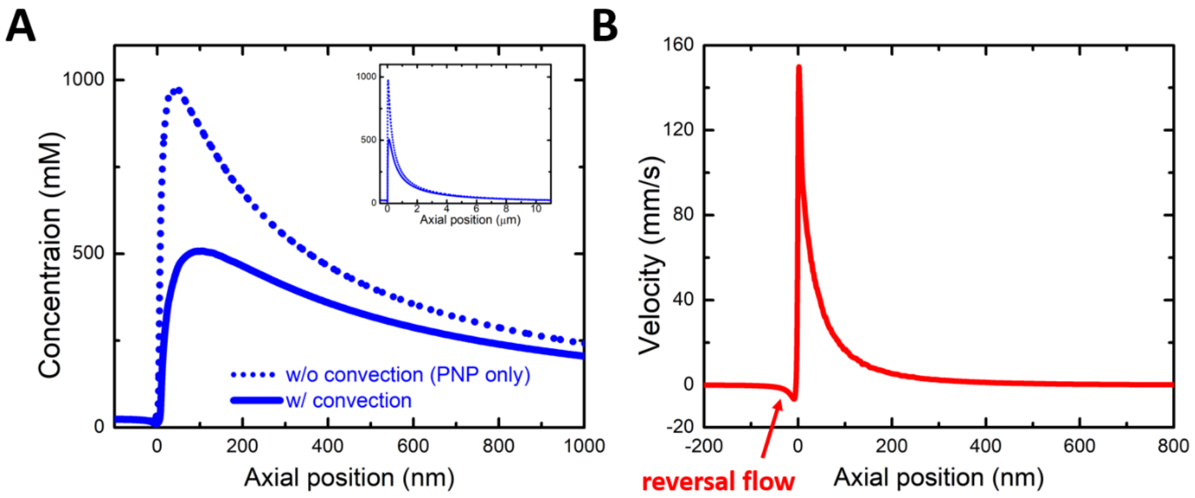

Figure S8. (A) Axial variation of total ionic concentration along the pore axis at 10 mM Cr^{3+}, 1 V, in the 4 nm in diameter nanopore. Solid curve: Model 2 with convection; dotted curve: Model 2 without convection. The inset shows the results for the whole membrane thickness of 11 μm. (B) Velocity profile along the pore axis for 10 mM Cr^{3+}, 1 V, and d_{tip} = 4 nm. Note the change of the velocity sign at the tip opening. Axial position of 0 denotes the location of tip opening.

References

(1) Kilic, M. S.; Bazant, M. Z.; Ajdari, A. Steric effects in the dynamics of electrolytes at large applied voltages. II. Modified Poisson-Nernst-Planck equations. *Phys. Rev. E* **2007**, *75* (2), 021503. DOI: 10.1103/PhysRevE.75.021503.
(2) Wang, H.; Thiele, A.; Pilon, L. Simulations of Cyclic Voltammetry for Electric Double Layers in Asymmetric Electrolytes: A Generalized Modified Poisson–Nernst–Planck Model. *J. Phys. Chem. C* **2013**, *117* (36), 18286-18297. DOI: 10.1021/jp402181e.
(3) Shklovskii, B. I. Screening of a macroion by multivalent ions: Correlation-induced inversion of charge. *Phys. Rev. E* **1999**, *60* (5), 5802-5811. DOI: 10.1103/PhysRevE.60.5802.
(4) Nguyen, T. T.; Grosberg, A. Y.; Shklovskii, B. I. Screening of a charged particle by multivalent counterions in salty water: Strong charge inversion. *J. Chem. Phys.* **2000**, *113* (3), 1110-1125. DOI: 10.1063/1.481890.
(5) Besteman, K.; Zevenbergen, M. A. G.; Lemay, S. G. Charge inversion by multivalent ions: Dependence on dielectric constant and surface-charge density. *Phys. Rev. E* **2005**, *72* (6), 061501. DOI: 10.1103/PhysRevE.72.061501.

(6) Lin, K. B.; Lin, C. Y.; Polster, J. W.; Chen, Y. F.; Siwy, Z. S. Charge Inversion and Calcium Gating in Mixtures of Ions in Nanopores. *J. Am. Chem. Soc.* **2020**, *142* (6), 2925-2934. DOI: 10.1021/jacs.9b11537.
(7) Davis, J. A.; Leckie, J. O. Surface Ionization and Complexation at Oxide-Water Interface .2. Surface Properties of Amorphous Iron Oxyhydroxide and Adsorption of Metal-Ions. *J. Colloid Interface Sci.* **1978**, *67* (1), 90-107. DOI: 10.1016/0021-9797(78)90217-5.
(8) Trefalt, G.; Behrens, S. H.; Borkovec, M. Charge Regulation in the Electrical Double Layer: Ion Adsorption and Surface Interactions. *Langmuir* **2016**, *32* (2), 380-400. DOI: 10.1021/acs.langmuir.5b03611.
(9) Healy, T. W.; White, L. R. Ionizable Surface Group Models of Aqueous Interfaces. *Adv. Colloid Interface Sci.* **1978**, *9* (4), 303-345. DOI: 10.1016/0001-8686(78)85002-7.
(10) Rabinowitz, J.; Edwards, M. A.; Whittier, E.; Jayant, K.; Shepard, K. L. Nanoscale Fluid Vortices and Nonlinear Electroosmotic Flow Drive Ion Current Rectification in the Presence of Concentration Gradients. *The Journal of Physical Chemistry A* **2019**, *123* (38), 8285-8293. DOI: 10.1021/acs.jpca.9b04075.
(11) Laohakunakorn, N.; Thacker, V. V.; Muthukumar, M.; Keyser, U. F. Electroosmotic Flow Reversal Outside Glass Nanopores. *Nano Lett.* **2015**, *15* (1), 695-702.
